# The 2-*C*-methylerythritol 4-phosphate pathway in melon is regulated by specialized isoforms for the first and last steps

**DOI:** 10.1093/jxb/eru275

**Published:** 2014-07-10

**Authors:** Montserrat Saladié, Louwrance P. Wright, Jordi Garcia-Mas, Manuel Rodriguez-Concepcion, Michael A. Phillips

**Affiliations:** ^1^Plant and Animal Genomics Programme, Institut de Recerca i Tecnologia Agroalimentàries and Centre for Research in Agricultural Genomics, CSIC-IRTA-UAB-UB, 08193 Barcelona, Spain; ^2^Department of Biochemistry, Max Planck Institute for Chemical Ecology, Beutenberg Campus, Hans Knöll Street 8, 07745 Jena, Germany; ^3^Plant Metabolism and Metabolic Engineering Programme, Centre for Research in Agricultural Genomics, CSIC-IRTA-UAB-UB, 08193 Barcelona, Spain

**Keywords:** 2-*C*-methylerythritol 4-phosphate, carotenoids, fruit development, isoprenoid biosynthesis, melon, metabolite profiling, phylogenetics, transcript profiling.

## Abstract

The 2-*C*-methyl-d-erythritol-4-phosphate (MEP) pathway provides the precursors for the biosynthesis of plastidial isoprenoids, which include the carotenoid pigments of many fruits. We have analysed the genes encoding the seven enzymes of the MEP pathway in melon (*Cucumis melo* L.) and determined that the first one, 1-deoxyxylulose 5-phosphate synthase (DXS), and the last one, 1-hydroxy-2-methyl-2-(*E*)-butenyl 4-diphosphate reductase (HDR), are represented in the genome as a small gene family and paralogous pair, respectively. In the case of DXS, three genes encode functional DXS activities which fall into previously established type I (*CmDXS1*) and II (*CmDXS2a* and *CmDXS2b*) categories, while a fourth DXS-like gene belonging to the type III group did not encode a protein with DXS activity. Their expression patterns and phylogenies suggest that *CmDXS1* is functionally specialized for developmental and photosynthetic processes, while *CmDXS2a* and *CmDXS2b* are induced in flowers and ripening fruit of orange- (but not white-) fleshed varieties, coinciding with β-carotene accumulation. This is the first instance connecting type II *DXS* genes to specialized isoprenoid biosynthesis in the fruit of an agronomically important species. Two HDR paralogues were shown to encode functional enzymes, although only *CmHDR1* was highly expressed in the tissues and developmental stages tested. Phylogenetic analysis showed that in cucurbits such as melon, these *HDR* paralogues probably arose through individual gene duplications in a common angiosperm ancestor, mimicking a prior division in gymnosperms, while other flowering plants, including apple, soy, canola, and poplar, acquired *HDR* duplicates recently as homoeologues through large-scale genome duplications. We report the influence of gene duplication history on the regulation of the MEP pathway in melon and the role of specialized MEP-pathway isoforms in providing precursors for β-carotene production in orange-fleshed melon varieties.

## Highlight text

This research describes the role of specialized isoforms encoding regulated steps of the MEP pathway in melon and provides evidence for their functional specialization in seedling greening.

## Introduction

Melon (*Cucumis melo* L.) is an economically important species in the family Cucurbitaceae, which also includes cucumber (*C. sativus*), squash (*Cucurbita spp*.), and watermelon (*Citrullus lanatus*). It is one of the most valuable fleshy fruits for fresh consumption in North America and Europe due to its distinct flavor and sugar content. Besides sweetness, the shelf life, colour, and aroma of melon are all important traits which have an impact on fruit quality. The latter two depend on the synthesis of isoprenoids, a ubiquitious family of natural compounds which play numerous central metabolic roles in all plants as precursors for protective pigments (carotenoids), hormones (brassinosteroids, gibberellins, strigolactones, cytokinins, and absiscic acid) and the side chains of chlorophylls, tocopherols, and quinone redox co-factors (Bouvier *et al.*, 2005).

In melon fruit, non-climacteric white-fleshed varietals such as ‘Piel de Sapo’ (PS) generally lack carotenoid pigments, whereas β-carotene, a nutritionally important carotenoid that serves as the main precursor for vitamin A synthesis, accumulates in climacteric orange-fleshed varieties such as ‘Vedrantais’ (Ved) and Dulce (Dul), resulting in their characteristic orange hue. These carotenoid-rich and -deficient melon strains provide a convenient comparative system for understanding the molecular regulation of carotenoid formation in fruit. However, an investigation of the upstream steps providing carotenoid precursors has not previously been undertaken.

Of the two pathways in plant cells which synthesize isoprenoid precursors, the 2-*C-*methyl-d-erythritol 4-phosphate (MEP) pathway is thought to be the main supplier of precursors for carotenoids (Rodríguez-Concepción, 2010). This pathway, as well as the cytosolic mevalonate pathway, provides the universal C_5_ intermediates isopentenyl and dimethylallyl diphosphate (IPP and DMAPP) which form the basis for all higher isoprenoid biosynthesis (Hunter, 2007; Phillips *et al.*, 2008; Cordoba *et al.*, 2009). In this pathway, pyruvate and glyceraldehyde 3-phosphate are condensed through the action of 1-deoxyxylulose 5-phosphate synthase (DXP synthase or DXS). Following the reductive isomerization of DXP to MEP by DXP reductoisomerase (DXR), it undergoes five additional enzymatic steps en route to the formation of IPP and DMAPP. The last of these steps is a reductive dehydroxylation to form both of these products from 1-hydroxy-2-methyl-2-(*E*)-butenyl 4-diphosphate (HMBPP) via HMBPP reductase (HDR).

DXS is typically encoded in plant nuclear genomes as a small gene family (Grassi *et al.*, 2013; Peng *et al.*, 2013). It is now well established that multiple clades of functionally specialized DXS classes exist in plants (Walter *et al.*, 2002). This distinction is based on phylogenetic analysis, biochemical characterization, and gene expression patterns and has been observed in angiosperm and gymnosperm plant lineages (Kim *et al.*, 2006; Phillips *et al.*, 2007; Vallabhaneni and Wurtzel, 2009; Cordoba *et al.*, 2011). Type I DXS genes are constitutively expressed in green tissues and are thought to supply the precursors for housekeeping and photosynthetic metabolites such as carotenoids and chlorophyll. Type II DXS genes are usually expressed in specialized contexts such as apocarotenoid-accumulating roots colonized by mycorrhizas (Walter *et al.*, 2000) or in the resin duct epithelial cells of conifers in response to bark beetle attack or fungal infection (Phillips *et al.*, 2007). A third clade of DXS-like sequences has recently been proposed (Cordoba *et al.*, 2011). DXS activity has not been definitively demonstrated for this group, but in maize, the expression of one member of this clade, *ZmDXS3*, correlates with endosperm carotenoid accumulation (Vallabhaneni and Wurtzel, 2009).

While the evolutionary origin of *DXS* from eubacterial sources has been investigated (Lange *et al.*, 2000; Krushkal *et al.*, 2003), the origin and subfunctionalization of DXS paralogues within plant genomes remains poorly understood. HDR, like DXS, is often encoded in plant genomes as multiple copies (Kim *et al.*, 2008; Kim *et al.*, 2009; Grassi *et al.*, 2013; Kang *et al.*, 2013), while the five intervening enzymes are encoded by singleton genes with few exceptions (Seetang-Nun *et al.*, 2008). Gene duplications leading to multiple copies of a gene may arise through several unrelated mechanisms, including retroposition, unequal crossing over, segmental or chromosomal duplications, and polyploidy (Zhang, 2003; Shoemaker *et al.*, 2006). Multiple models for the retention and evolution of gene duplicates have been proposed (Innan and Kondrashov, 2010), ultimately culminating in functional specialization (Veitia *et al.*, 2008), pseudogenization, or deletion.

We have taken advantage of the recently published melon genome (Garcia-Mas *et al.*, 2012) to determine whether the genome architecture of MEP-pathway paralogues in melon could help explain the complex process of fruit carotenoid biosynthesis. We report that small-scale gene duplications in the MEP pathway have led to specialized isoforms in melon which play an important role in providing carotenoid precursors during fruit ripening, including the first report of type II DXS proteins involved in this process.

## Materials and methods

### Plant material

Tissue from three different melon varieties were used: the non-climacteric/white flesh melon type Piel de Sapo (PS) from the *inodorus* group and the climacteric/orange flesh types Vedrantasi (Ved) and Dulce (Dul) from the *cantalupensis* and *reticulatus* groups, respectively. Plants were grown under the same greenhouse conditions as previously described (Eduardo *et al.*, 2005). Flowers (one per plant) were hand pollinated to control fruit stages. Fruits were collected at four different stages covering fruit development and ripening [15, 25, and 35 days after pollination (DAP) and harvest; 45 DAP for Ved and 55 DAP for PS]. Flesh-mesocarp from three different fruit replicates of each stage was extracted from the middle of the fruit, avoiding rind, seeds, and jelly, and then frozen immediately in liquid nitrogen and stored at –80°C. Vegetative tissues (leaves, flowers, stems, and roots) were also frozen in liquid nitrogen and kept at –80°C. For light-induction experiments, Ved seeds were germinated in the dark for 4 days, transferred to the light (120 μEinstein∙m^–2^∙s^–1^) for 4, 8, 12, or 24h and then flash frozen in liquid nitrogen.

### Gene expression analysis by quantitative RT-PCR

Total RNA samples were extracted using the Qiagen (Hilden, Germany) RNeasy Plant Mini Kit^®^. RNA quantity and quality were evaluated using a NanoDrop ND-1000 spectrophotometer (Thermo Scientific, Waltham, MA, USA) and assessed by agarose gel electrophoresis. RNA was then treated with RNAse free TURBO-DNase I (Turbo-DNA-*free* ^TM^ Kit; Life Technologies, Ambion^®^, Carlsbad, CA, USA) for 30min at 37°C before being used as a template for cDNA synthesis. First-strand cDNA synthesis was performed using 1 μg total RNA, Superscript^TM^ III First Strand Synthesis (Invitrogen Life Technologies, Carlsbad), and an oligo(dT)_20_ primer according to the manufacturer’s instructions. The synthesized cDNA was diluted 1:5 with pure water, and 2 μl were used in subsequent reactions. Quantitative RT-PCR (QPCR) assays were performed on a LightCycler^®^ 480 Real-Time PCR System (Roche Applied Science, Penzberg, Germany) using SYBR^®^ Green I Mix (Roche Applied Science). Relative transcript levels of DXS and HDR isoforms were calculated using the primer efficiency-corrected relative quantification model (Pfaffl, 2001) to compare a single gene in different tissues or the ΔΔCt model to compare multiple genes in light induction or fruit development. In these cases, relative transcript levels were calculated using the median gene expression of all DXS and HDR genes surveyed. cDNA loading was normalized using three reference genes: cyclophilin (*CmCYP7*; MELO3C025848) (Gonzalez-Ibeas *et al.*, 2007; Mascarell-Creus *et al.*, 2009), adenine phosphoribosyltransferase *(CmAPT;* MELO3C013962), and actin 2/8 (*CmACT*; MELO3C023264). Primers for amplification of target and reference genes were designed with Primer Express^®^ Software v2.0 (Applied Biosystems, Foster City, CA, USA). Primer sequences are listed in Supplementary Table 1. Intra-assay variation was evaluated by performing all amplification reactions in triplicate. The amplification protocol consisted of an initial step at 95°C for 10min, and 45 cycles at 95°C for 10 s and 60°C for 30 s. The specificity of the PCR amplification was confirmed by melting curve analysis and by sequencing of the PCR amplicons. Reverse transcriptase minus controls (RT–) were performed once for each RNA sample to determine the amount of contaminating genomic DNA, and non-template controls (NTC) were included in each plate to monitor the presence of DNA contamination and the formation of primer–primer dimers.

### Phylogenetic analysis of DXS and HDR proteins

HDR and DXS protein sequences were downloaded from the NCBI database. Where indicated, sequences were obtained from genome-sequencing databases. Putative chloroplast transit peptide cleavage points were calculated using the ChloroP 1.1 Server (Emanuelsson *et al.*, 1999) and after manual curation, they were excluded for the purposes of sequence analysis. The phylogenetic analysis was performed on the Phylogeny.fr platform (www.phylogeny.fr). Sequences were aligned with MUSCLE (v3.7) with default settings. The phylogenetic tree was reconstructed using the maximum likelihood method implemented in the PhyML program (v3.0 aLRT). The default substitution model was selected assuming an estimated proportion of invariant sites of 0 and 4 gamma-distributed rate categories to account for rate heterogeneity across sites. The gamma shape parameter was estimated directly from the data (gamma = 0.769). Reliability for internal branches was assessed using the aLRT test (SH-like) (Anisimova and Gascuel, 2006). Graphical representation and editing of the phylogenetic trees were performed with Interactive Tree of Life (iTOL) (Letunic and Bork, 2010). For SNP analysis of coding and *cis*-regulatory regions, the gene sequences of CmDXS2a, CmDXS2b, and CmHDR1 from the PS and Ved varieties, including the promoter (2kb upstream of the start codon) and 3ʹUTR (1kb downstream of the stop codon), were retrieved from their previously available re-sequenced genomes (Sanseverino *et al.*, unpublished) after mapping reads against the DHL92 melon reference genome (Garcia-Mas *et al.*, 2012). SNP and short indels were extracted with SUPER v 0.9 (Sanseverino *et al*, unpublished) using the algorithms of BWA (Burrows-Wheeler Aligner) (Li and Durbin, 2010) (http://bio-bwa.sourceforge.net) and SAMtools (Sequence Alignment/Map) (Li *et al.*, 2009) (http://samtools.sourceforge.net) using a read depth >10 and a base-calling quality cutoff >30. Predicted transcriptional start sites and differences in transcription factor binding sites between PS and Ved were determined for these three genes using the PLACE database (Higo *et al.*, 1999) (http://www.dna.affrc.go.jp/PLACE/).

### HPLC analysis

The isoprenoid pigment content of the melon fruit samples was examined by HPLC-DAD-FLD. Except where noted, all chemical reagents were obtained from Sigma-Aldrich (St. Louis, MO, USA). All samples were protected from light and heat during all steps of the extraction procedure. 5mg lyophilized melon tissue were extracted with agitation for 15min at 4°C with 1ml chilled hexane:acetone:methanol (2:1:1) containing 6 μg canthaxanthin·ml^–1^, followed by the addition of 100 μl water and further extraction for 20 s. Samples were centrifuged at maximum speed in a chilled benchtop microcentrifuge (Eppendorf 5418R, Hamburg, Germany) for 3min. The upper organic phase was aspirated and set aside while the pellet was extracted a second time with the same solvent lacking canthaxanthin. Following the same extraction and centrifugation procedure, the organic phases were pooled and dried in a vacuum centrifuge (Eppendorf Concentrator Plus). Dried residues were resuspended in 150 μl ethyl acetate and filtered through a 0.2 μm PTFE microfilter (Supelco). A 10 μl aliquot of each sample was injected onto an Agilent Technologies (Santa Clara, CA, USA) 1200 series HPLC system equipped with a diode array and a 250mm × 4.6mm C30 reverse phase column (YMC, Kyoto, Japan) equilibrated with methanol:water (99:1) containing 0.2% ammonium acetate (solvent A). Metabolites were separated with the following gradient: isocratic separation in 100% solvent A, followed by a step to 15% solvent B (*tert* butyl methyl ether) at 12min and a linear gradient to 100% B by 38min. Following a 6min hold at 100% B, the column was returned to initial conditions and equilibrated for an additional 6min. The flow rate was maintained at 1 ml·min^–1^. Isoprenoid pigments were monitored at 472 and 650nm and compared to the retention times of chlorophyll and β-carotene authentic standards. Tocopherol quantification was accomplished through fluorescence detection (excitation 290nm, emission 330nm) and comparison to a standard curve constructed from α-tocopherol.

### Cloning DXS and HDR isoforms

In order to verify enzymatic activity of DXS and HDR gene family members, a combination of *in vitro* enzyme assays and bacterial complementation assays were carried out. In both cases, the corresponding genes bearing plastidial transit peptides were PCR amplified without their transit peptides to improve solubility in heterologous expression systems. All cloning steps were carried out using the Gateway^TM^ cloning system (Invitrogen Life Technologies). Sequences of the corresponding attB1 and attB2 primers used for each DXS or HDR gene are listed in Supplementary Table 1. Truncated amplicons lacking the portions of the full-length coding sequences encoding transit peptides were thus amplified bearing attB adaptors. The PCR programme consisted of a 3min denaturation step at 95°C followed by four cycles of low-stringency PCR (96°C for 10 s, 48°C for 45 s, and 72°C for 2min). This priming stage was followed by 32 cycles of PCR as follows: 96°C for 10 s, 63°C for 45 s, and 72°C for 2min. The 50 μl PCRs included 1 μl pooled cDNA from various Ved organs, 0.25 μl AccuPrime^TM^ high fidelity TAQ (Invitrogen Life Technologies), 10 pmol attB1 and attB2 primer, and 1X AccuPrime^TM^ buffer I. PCRs were examined on 2% agarose gels, and reactions with a single product of expected size were used without further purification in a 10 μl BP clonase II reaction (Invitrogen Life Technologies) containing ~150ng PCR product and 150ng pDONR207 entry vector. Following an overnight reaction at room temperature, recombined products were transformed into TOP10 chemically competent cells by heat shock and plated onto LB media containing 50 μg·ml^–1^ gentamycin. Colonies were selected and grown in liquid media containing the same antibiotic. Plasmid DNA was purified using the QIAprep Spin Miniprep kit (QIAGEN). Multiple inserts of each gene were fully sequenced and compared to the published melon genome sequence. Purified plasmid DNA was diluted to 150 ng·ml^–1^ and used in an LR clonase II recombination reaction (Invitrogen Life Technologies) with either a Gateway-ready version of the pET28 (for *in vitro* enzyme assays) or pET32 (for bacterial complementation) plasmids (Clontech Laboratories, Mountain View, CA, USA), transformed as before, and plated on LB media containing either carbenicillin at 100 μg·ml^–1^ (pET32) or kanamycin at 50 μg·ml^–1^ (pET28). The resulting transformants were grown in 25ml LB media containing the appropriate antibiotic and purified as described above to generate stocks of each gene in expression vectors.

### Bacterial complementation

The *Escherichia coli* HDR mutant strain MG1655 *ara<>ispH* (McAteer *et al.*, 2001) was used to verify activity of CmHDR1 and CmHDR2 as described previously (Hsieh and Goodman, 2005). The *ara<>ispH* strain was maintained on LB media containing 50 μg·ml^–1^ kanamycin and 0.2% (w/v) arabinose. A modifed pET32 vector (Yu and Liu, 2006) bearing either melon *CmHDR1* or *CmHDR2* was introduced into competent *ara<>ispH* cells and selected on LB plates containing 0.2% arabinose, 50 μg·ml^–1^ kanamycin, and 100 μg·ml^–1^ carbenicillin. Colonies were transferred to plates containing the same antibiotics but 0.2% (w/v) glucose in place of arabinose to suppress the expression of endogenous HDR activity in this modified strain. As controls, the same strain was transformed with the pQE30 plasmid containing *E. coli HDR* (positive control) and pET32 containing spruce DXS1 (negative control).

DXS activity was first evaluated using a *dxs* deficient strain of *E. coli* which had been engineered to synthesize isopentenyl and dimethylallyl diphosphate (IPP and DMAPP) from mevalonate (Campos *et al.*, 2001). Competent cells grown in the presence of 1mM mevalonate were transformed with purified pET32 destination vectors containing a candidate cDNA (*CmDXS1*, *CmDXS2a*, *CmDXS2b*, *CmDXS3/DXL*, or control) and plated on LB media containing 1mM mevalonate and 100 μg·ml^–1^ carbenicillin. To test for a functional DXS gene, transformed colonies were transferred to LB-carbenicillin plates lacking mevalonate and incubated at 37°C overnight.

### *In vitro* DXS enzyme assay

CmDXS3/DXL activity was further examined by *in vitro* assay following expression in *E. coli* strain BL21 Rosetta (Novagen Biosciences, Madison, WI, USA) using the pET28 vector. Following transformation of competent cells, 10ml cultures of OverNight Express media (Novagen Biosciences) containing 10ml glycerol·l^–1^ and 50 μg·ml^–1^ kanamycin were inoculated with a single colony and grown for 48h at 28°C. Stationary cultures were harvested by centrifugation at 2000*g* for 20min at 4°C. The pellet was resuspended in 1mL 50mM Tris pH 8.0 containing 5% (v/v) glycerol, 50mM KCl, 25mM MgCl2, 1mM ascorbic acid, 100 μM thiamin pyrophosphate (TPP), 0.2% (v/v) Tween-20, and bacterial protease inhibitor cocktail (Sigma Aldrich) diluted 1:100. The resuspended pellet was subjected to two freeze-thaw cycles using ethanolic ice and a water bath and then sonicated using a Branson Digital Sonifier (6min total, at 25% intensity, with a 1 s duty cycle and 1 s rest between pulses). The lysed cultures were centrifuged at 16 000*g* and 4°C, and the supernatants were filtered through a 0.45 μm acetate syringe filter. Protein concentrations were judged by comparison to a BSA standard curve constructed using Bradford reagent according to the manufacturer’s instructions (Bio-Rad Laboratories, Hercules, CA, USA). All protein samples were adjusted to 0.5 mg·ml^–1^. A 30 μl aliquot of each crude protein sample was combined with 70 μl DXS reaction buffer. This buffer was identical to the resuspension buffer except it contained TPP at 1mM and sodium pyruvate and glyceraldehyde 3-phosphate at 14.28mM each (final concentrations 10mM). Upon combining purified proteins with reaction buffer, the reactions were incubated at 25°C for 2h. Reactions were halted by addition of 100 µl CHCl_3_, followed by 10 s vigorous vortexing and 1min centrifugation. DXP in the upper aqueous fraction was analysed by LC-MSMS as described in Wright and Phillips (2014).

## Results

### Identification of MEP pathway genes in melon

The MEP pathway provides precursors for isoprenoid biosynthesis and has an impact on important fruit traits such as carotenoid production in melon. Based on a search of the recently published melon genome using orthologous sequences from other plant species, we identified and cloned the full complement of MEP-pathway genes (Table 1). Individual sequencing of isolated cDNAs from the orange-fleshed Ved variety indicated that all MEP pathway genes matched their sequences with the melon reference genome DHL92. The white-fleshed PS variety, in which the fruit does not produce carotenoids, was included in our analysis for comparative purposes. Whole genome re-sequencing of the same coding sequences from PS confirmed a near-identical correspondence at the nucleotide level and no amino acid differences in MEP-pathway enzymes compared to Ved. Of the seven steps in the pathway, five are catalysed by enzymes encoded by single-copy genes in melon, whereas multiple isoforms code for the first and last enzymes of the pathway, DXS and HDR, respectively (Table 1). Based on ChloroP analysis of their deduced amino acid sequences, all the encoded proteins contain a transit peptide which directs the pseudomature protein to the plastid.

**Table 1.** Melon genes of the MEP pathway

Gene	MELOGEN ID	Full length (aa)	Mature length (aa)^b^	% similarity to *Arabidopsis* orthologue^c^	Genome scaffold and linkage group
*CmDXS1*	MELO3C021375	718	655	89	CM3.5_scaffold00047 (XI)
*CmDXS2a*	MELO3C017499	732	672	72	CM3.5_scaffold00030 (II)
*CmDXS2b*	MELO3C014965	721	687	75	CM3.5_scaffold00023 (VI)
*CmDXS2b’*	MELO3C014966/3C014967	665^a^	628	64	CM3.5_scaffold00023 (VI)
*CmDXS3/DXL*	MELO3C011230	668	625	60	CM3.5_scaffold00014 (III)
*CmDXR*	MELO3C026292	469	401	90	CM3.5_scaffold00089 (II)
*CmMCT*	MELO3C016431	304	241	74	CM3.5_scaffold00028 (VI)
*CmCMK*	MELO3C015605	394	347	78	CM3.5_scaffold00025 (II)
*CmMDS*	MELO3C016339	230	182	86	CM3.5_scaffold00027 (VII)
*CmHDS*	MELO3C005061	741	697	88	CM3.5_scaffold00004 (XII)
*CmHDR1*	MELO3C018407	463	415	82	CM3.5_scaffold00034 (I)
*CmHDR2*	MELO3C021949	466	419	76	CM3.5_scaffold00050 (XI)

^a^ Estimated from a reconstruction of the coding sequence. ^b^ Estimated from ChloroP predictions and multiple sequence alignments. ^c^ Putative transit peptides were excluded from this calculation. aa, amino acids.

### Only three of the five predicted melon *DXS* genes encode functional DXS enzymes

**Fig. 1.**
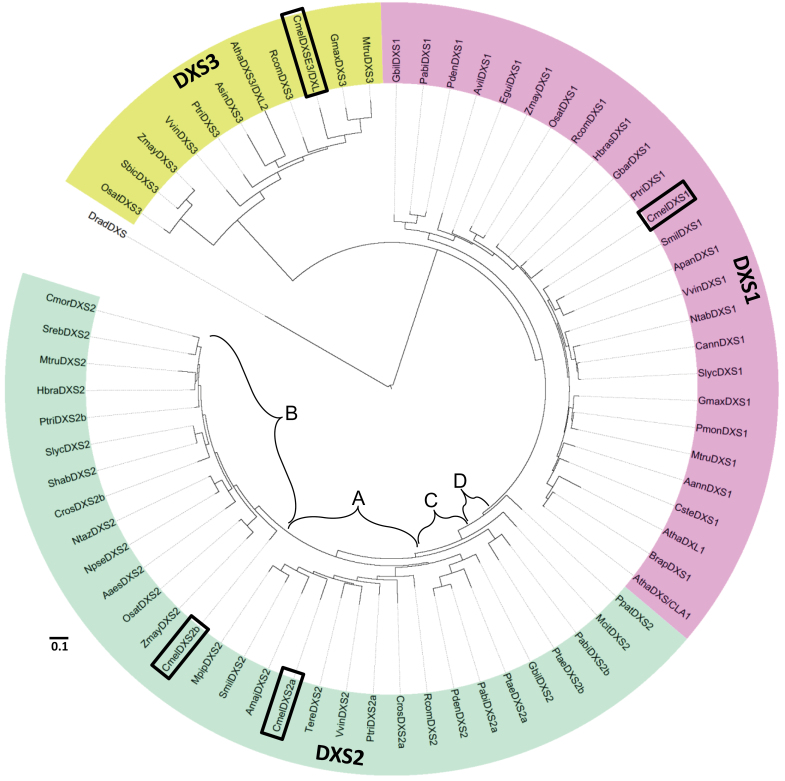
Phylogenetic analysis of DXS protein sequences. Sequences were aligned with MUSCLE (v3.7). Construction of the phylogenetic tree was based on the maximum likelihood method of the PhyML program (v3.0 aLRT). Graphical representation and edition of the phylogenetic tree were performed with Interactive Tree of Life (iTOL). DXS1, DXS2 and DXS3 clades are represented in purple, green, and yellow, respectively. A and B signify angiosperm type II subclasses, while C and D represent similar subclasses for gymnosperm type II proteins. Melon DXS sequences are boxed. Internal branching reliability (values not shown) was established using the aLRT test. The sequences used in the tree were as follows: AaesDXS2: ABK35283.1 *Adonis aestivalis* var. palaestina; AannDXS1: AAD56390.2 *Artemisia annua*; AmajDXS2: AAW28999.1 *Antirrhinum majus*; ApanDXS1: AAP14353.1 *Andrographis paniculata*; AsinDXS3: AFU75320.1 *Aquilaria sinensis*; AthaDXL1: At3g21500 *Arabidopsis thaliana*; AthaDXS/CLA1: At4g155560 *A. thaliana*; AthaDXS3/DXL2: At5g11380 *A. thaliana*; AvilDXS1: ACR02668.1 *Amomum villosum;* BrapDXS1: ABE60813.1 *Brassica rapa*; CannDXS1: Y15782 *Capsicum annuum*; CmelDXS1: MELO3C011230 *Cucumis melo*; CmelDXS2a: MELO3C017499 *C. melo*; CmelDXS2b: MELO3C014965 *C. melo*; CmelDXS3/DXL: MELO3C021375 *C. melo*; CmorDXS2: BAE79547.1 *Chrysanthemum × morifolium*; CrosDXS2a: CAA09804.2 *Catharanthus roseus;* CrosDXS2b: ABI35993.1 *C. roseus*; CsteDXS1: BAF75640.1 *Croton stellatopilosus*; DradDXS (outgroup): NP_295198 *Deinococcus radiodurans;* EguiDXS1: AAS99588.1 *Elaeis guineensis*; HbraDXS1: AAS94123.1 *Hevea brasiliensis*; HbraDXS2: ABF18929.1 *H. brasiliensis*; GbarDXS1: ABN13970.1 *Gossypium barbadense*; GbilDXS1: AAS89341.1 *Ginkgo biloba*; GbilDXS2: AAR95699.1 *G. biloba*; GmaxDXS1: ACO72582.1 *Glycine max*; GmaxDXS3: XP_003527796.1 *G. max*; McitDXS2: AAL32062.1 *Morinda citrifolia*; MpipDXS2: AAC33513.1 *Mentha × piperita*; MtruDXS1: CAD22530 *Medicago truncatula*; MtruDXS2: CAN89181.1 *M. truncatula*; MtruDXS3: XP_003603440.1 *Medicago truncatula*; NpseDXS2: CAC08458.1 *Narcissus pseudonarcissus*; NtabDXS1: CBA12009.1 *Nicotiana tabacum*; NtazDXS2: ADD82535.1 *Narcissus tazetta* var. Chinensis; OsatDXS1: NP_001055524.1 *Oryza sativa* Japonica; OsatDXS2: NP_001059086.1 *O. sativa* Japonica; OsatDXS3: BAA83576 *O. sativa* Japonica; PabiDXS1: ABS50518 *Picea abies*; Pabi DXS2a: ABS50519 *P. abies*; PabiDXS2b: ABS50520 *P. abies*; PdenDXS1: ACC54557.1 *Pinus densiflora*; PdenDXS2: ACC54554.1 *P. densiflora*; PmonDXS1: AAQ84169.1 *Pueraria montana* var. Lobata; PpatDXS2: XP_001756357.1 *Physcomitrella patens* subsp. patens; PtaeDXS2a: ACJ67020.1 *Pinus taeda;* PtaeDXS2b: ACJ67021.1 *P. taeda* (originally annotated as type I DXS); PtriDXS1: XP_002312717.1 *Populus trichocarpa*; PtriDXS2a: XP_002331678.1 *P. trichocarpa*; PtriDXS2b: XP_002303416.1 *P. trichocarpa*; PtriDXS3: XP_002308644.1 *P. trichocarpa*; RcomDXS1: XP_002516843.1 *Ricinus communis*; RcomDXS2: XP_002533688.1 *R. communis*; RcomDXS3: XP_002514364.1 *R. communis*; SbicDXS3: XP_002437810.1 *Sorghum bicolour*; ShabDXS2: AY687353 *Solanum habrochaites*; SmilDXS1: ACF21004.1 *Salvia miltiorrhiza*; SmilDXS2: ACQ66107.1 *S. miltiorrhiza*; SlycDXS1: CAZ66648.1 *Solanum lycopersicum*; SlycDXS2: CAZ66649.1 *S. lycopersicum*; SrebDXS2: AJ429232 *Stevia rebaudiana*; TereDXS2: AF251020 *Tagetes erecta*; VvinDXS1: XP_002271585.1 *Vitis vinifera*; VvinDXS2: XP_002271782.2 *V. vinifera*; VvinDXS3: XP_002277919.1 *V.* vinifera; ZmayDXS1: ACG27905.1 *Zea mays*; ZmayDXS2: DAA59892.1 *Z. mays*; ZmayDXS3: ABP88135.1 *Z. mays*. A class number was appended to sequences not originally annotated according to DXS class (type I, II, or III) according to the clade where they group in our analysis. Species with multiple type 2 DXS sequences are referred to here as DXS2a and DXS2b if not originally annotated as such. Bar, 0.1 amino acid substitutions per site.

Four full-length sequences showing similarity to DXS were found in the melon genome (Table 1). Construction of a phylogenetic tree which includes these four DXS candidates as well as other known plant DXS genes indicated that only one melon sequence grouped with the type I DXS clade (Fig. 1) and was denoted *CmDXS1*. Two grouped with type II sequences, hereafter referred to as *CmDXS2a* and 2b. *CmDXS2a* and 2b did not form their own clade amongst other angiosperm type II sequences and instead fell into distinct subclasses of type II DXS (Fig. 1, clades A and B). The hierarchical structure of the type II clade suggests multiple rounds of DXS gene duplication have taken place in plants. For example, a similar subdivision among gymnosperm type II proteins is evident (Fig. 1, clades C and D). However, this hierarchy also indicates that type II gymnosperm proteins of clade C are more closely related to angiosperm sequences of clade A than to their own paralogues (group D). The DXS type II B clade includes both mono- and dicotyledonous species and is distinct from the common branch which yields the A and C clades, consisting of angiosperm and gymnosperm sequences, respectively. Excluding the transit peptide, the two melon type II sequences share 76% amino acid identity (90% similarity) while CmDXS1 shares 72 and 75% with CmDXS2a and 2b, respectively (86 and 87% similarity). The fourth DXS candidate grouped with a third clade of DXS-like sequences (type III; Fig. 1) with largely unknown functions and was designated CmDXS3/DXL (DXS-like). Its deduced amino acid sequence possesses 60, 55, and 57% identity to CmDXS1, 2a, and 2b, respectively (76, 74, and 73% similarity).

A fifth DXS candidate initially identified as two separate coding sequences by automatic genome annotation (MELO3C014966 and MELO3C014967) is apparently the product of tandem gene duplication of *CmDXS2b* and is located directly adjacent to it (*CmDXS2bʹ*). However, it has been interrupted by an LTR transposable element (position 5573690-5577498 of the reference genome), and quantitative RT-PCR assays using specific primers failed to detect the presence of transcripts in any tissue or developmental stage (data not shown). From this we conclude *CmDXS2bʹ* to be a non-functional gene. This transposon insertion was present in both Ved and PS melon types.

**Fig. 2.**
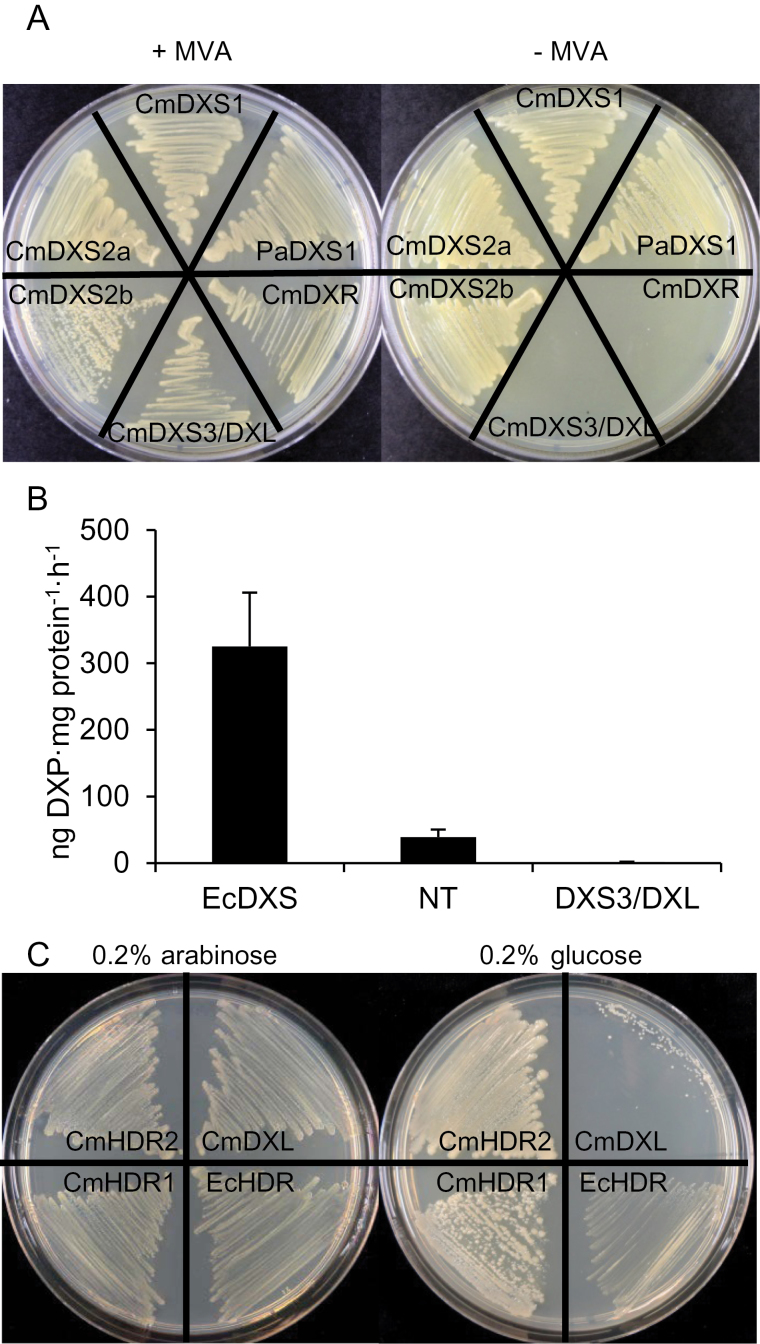
Confirmation of enzyme activity in melon DXS and HDR genes. (A) Complementation of a *dxs- E. coli* mutant with melon DXS isoforms. The *dxs–* strain can grow normally in the presence of 1mM mevalonate but requires transformation with a plasmid encoding a functional DXS gene for viability on mevalonate-free media. *Picea abies* DXS1 (PaDXS1) is included as a positive control (Phillips *et al.*, 2007) while melon 1-deoxyxyulose 5-phosphate reductoisomerase (CmDXR) was used as a negative control. (B) Crude lysates of *E. coli* overnight cultures expressing CmDXS proteins were used in DXS *in vitro* reactions with pyruvate, glyceraldehyde 3-phosphate, and all necessary co-factors. DXP was analysed by LC-MS/MS. The non-transformed control (NT) indicates endogenous DXS levels. (C) complementation of the *hdr E. coli* mutant *MG1655 ara*<>*ispH* (McAteer *et al.*, 2001) with melon HDR isoforms. The LB-carbenicillin-kanamycin plate on the left also contains 0.2% arabinose, which induces expression of the endogenous HDR gene in this mutant strain. The plate on the right contains the same antibiotics and 0.2% glucose, which represses endogenous HDR expression. Under these conditions, the strain is viable only when transformed with a plasmid encoding a functional HDR gene.

To verify the predicted enzymatic function for the four full-length candidates, we employed a *dxs*-deficient mutant strain of *E. coli* that had been engineered to convert mevalonate into IPP and DMAPP (Campos *et al.*, 2001). When transformed with a plasmid containing a functional DXS gene, the strain can grow without mevalonate. Thus, when this strain was transformed with a pET32 plasmid encoding Norway spruce DXS1 (Phillips *et al.*, 2007) as a positive control, *CmDXS1*, *CmDXS2a*, or *CmDXS2b* transformants grew in the absence of mevalonate (Fig. 2A). However, no growth was observed on mevalonate-free media when the same strain was transformed with melon DXR (negative control). The *dxs* mutant also failed to grow on media lacking mevalonate when transformed with pET32 bearing *CmDXS3/DXL* (Fig. 2A). An additional attempt to detect DXS activity for this protein was made using a highly sensitive *in vitro* LC-MS/MS based DXS assay which detects DXP formation directly. *CmDXS3/DXL* was subcloned without its predicted transit peptide into the pET28 vector for expression in *E. coli.* Cultures harbouring the same plasmid encoding the native *E.coli* DXS or non-transformed cells were used as positive and negative controls, respectively. Crude cell-free protein extracts of cultures overexpressing *E. coli* DXS demonstrated high levels of DXP formation under optimized *in vitro* assay conditions (Fig. 2B), while non-transformed crude extracts produced a lower but easily quantifiable level of DXP due to endogenous DXS activity. Surprisingly, total protein extracts of cultures expressing CmDXS3/DXL produced no detectable DXP under the same conditions. One possible explanation for this apparent inhibitory effect could be that CmDXS3/DXL catalyses a distinct but related reaction which may involve one of the two substrates required by DXS. Given the lack of demonstrable DXS activity, we refer to CmDXS3/DXL hereafter as CmDXL (for DXS-like protein), following previous nomenclature recommendations (Phillips *et al.*, 2008).

### Two genes encode functional HDR enzymes in melon

**Fig. 3.**
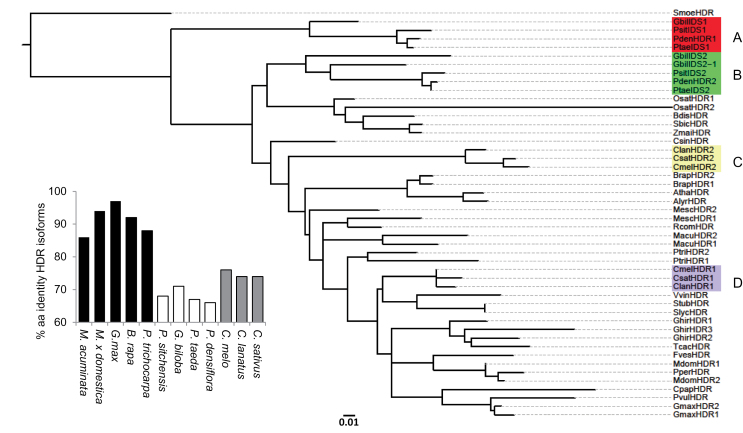
Phylogenetic analysis of plant HDR protein sequences. Sequences were aligned with MUSCLE (v3.7). Construction of the phylogenetic tree was based on the maximum likelihood method of the PhyML program (v3.0 aLRT). Graphical representation and edition of the phylogenetic tree were performed with Interactive Tree of Life (iTOL, itol.embl.de). Internal branching reliability (values not shown) was established using the aLRT test. Gymnosperm HDR1 (A) and HDR2 (B) clades are displayed in red and green, respectively. Cucurbit HDR2 (C) and HDR1 (D) clades are displayed in yellow and purple, respectively. For those sequences not available on NCBI, the protein code in bold corresponds to the genome sequence annotation. The sequences used in the tree were as follows: AthaHDR: NP_567965 *Arabidopsis thaliana*; AlyrHDR: XP_002867122 *A. lyrata*; BdisHDR: XP_003560190 *Brachypodium distachyon*; BrapHDR1: **Bra034620** *Brassica rapa*; BrapHDR2: **Bra011522** *B. rapa*; ClanHDR1: **Cla015963** *Citrullus lanatus*; ClanHDR2: **Cla010297** *C. lanatus*; CmelHDR1: **MELO3C018407P1** *Cucumis melo*; CmelHDR2: **MELO3C021949P1** *C. melo*; CpapHDR: **CP00062G01010** *Carica papaya*; CsatHDR1: XP_004162740 *Cucumis sativus*; CsatHDR2: XP_004145400 *C. sativus*; CsinHDR: **orange1.1g016939m** *Citrus sinensis*; FvesHDR: XP_004289689 *Fragaria vesca*; GhirHDR1: **Gorai.008G019000.1** *Gossypium hirsutum*; GhirHDR2: **Gorai.005G229100.1** *G. hirsutum*; GhirHDR3: **Gorai.013G052300.1** *G. hirsutum*; GbilIDS1: ABB78088 *Ginkgo biloba*; GbilIDS2: ABB78090 *G. biloba*; GbilIDS2-1: ABB78089 *G. biloba*; GmaxHDR1: XP_003537898 *Glycine max*; GmaxHDR2: XP_003541082 *G. max*; MacuHDR1: **GSMUA_Achr8P04900_001** *Musa acuminata*; MacuHDR2: **GSMUA_Achr3P25710_001** *M. acuminata*; MdomHDR1: **MDP0000762895** *Malus domestica*; **MdomHDR2: MDP0000029783** *M. domestica*; MescHDR1: **cassava4.1_007197m** *Manihot esculenta*; MescHDR2: **cassava4.1_007171m** *M. esculenta*; OsatHDR1: NP_001051167 *Oryza sativa*; OsatHDR2: NP_001051168; PsitIDS1: ACN40284 *Picea sitchensis*; PsitIDS2: ACN39959 *P. sitchensis*; PdenHDR1: ACC54560 *Pinus densiflora*; PdenHDR2: ACC54561 *P. densiflora*; PperHDR: EMJ01027 *Prunus persica*; PtaeIDS1: ABO26587 *Pinus taeda*; PtaeIDS2: ABO26588 *P. taeda*; PtriHDR1: XP_002305413 *Populus trichocarpa*; PtriHDR2: XP_002313816 *P. trichocarpa;* PvulHDR: **Pvul.011G047900.1** *Phaseolus vulgaris*; RcomHDR: XP_002519102 *Ricinus communis*; SbicHDR: **Sb01g009140.1** *Sorghum bicolour*; SlycHDR: NP_001234728 *Solanum lycopersicum*; SmoeHDR (outgroup): XP_002967048 *Selaginella moellendorffii*; StubHDR: **PGSC0003DMP400043678** *Solanum tuberosum*; TcacHDR: EOX98548 *Theobroma cacao*; VvinHDR: XP_002284659 *Vitis vinifera*; ZmaiHDR: NP_001169300 *Zea mays*. Bar, 0.01 amino acid substitutions per site. Inset, amino acid identity between full-length HDR homoeologues (black) from polyploid angiosperms, HDR paralogues from gymnosperms (white), or HDR paralogues from cucurbits (grey).

Two sequences showing similarity to known plant HDRs were found in the melon genome and designated *CmHDR1* and *CmHDR2* (Table 1). The melon HDR1 and HDR2 proteins share 78% amino acid identity and 90% similarity (excluding transit peptides). A phylogenetic analysis of HDR genes across the plant kingdom shows that the presence of multiple copies in a genome is a fairly common occurrence (Fig. 3). Unlike the well separated clades in the tree constructed from DXS protein sequences (Fig. 1), the phylogenetic distribution of HDR sequences is less evident. In species with multiple HDR genes, we detected two separate patterns. The first includes angiosperms with two HDR isoforms which resemble each other more than orthologous sequences from related species (Fig. 3, inset). For example, banana, apple (Velasco *et al.*, 2010), soybean (Schmutz *et al.*, 2010), canola (Wang *et al.*, 2011), and poplar each possess two HDR genes which form a species-specific subclade relative to their nearest neighbours. Each of these species has undergone at least one recent whole-genome duplication (WGD) event (Parkin *et al.*, 2003; Tuskan *et al.*, 2006; Schlueter *et al.*, 2007; Han *et al.*, 2011; D’Hont *et al.*, 2012). This fact, coupled to the high degree of similarity between paralogous pairs, suggests these HDR isoforms may be homoeologues produced by polyploidy. The second pattern, which includes spruce, ginkgo, pine, melon, watermelon, and cucumber, features separate HDR classes in which member similarity is higher than the similarity between paralogous pairs within a species. Gymnosperm (Fig. 3A and B) and cucurbit (Fig. 3C and D) HDR paralogues formed independent subclades each consisting of type I and type II isoforms. Interestingly, none of the genomes in this second group has undergone recent genome duplication events (Ahuja, 2005; Huang *et al.*, 2009; Garcia-Mas *et al.*, 2012; Guo *et al.*, 2013; Nystedt *et al.*, 2013). The lack of polyploidy or partial genome duplications among these species and the greater phylogenetic distance between their paralogues, in contrast to the first group, implies such paralogues may have arisen through individual gene duplications.

The activity of both melon HDR isoforms was affirmed using a bacterial complementation assay. The *MG1655 ara*<>*IspH* mutant strain expresses an endogenous HDR under the control of the inducible araBAD promoter, which is alternately induced by arabinose or repressed by glucose (McAteer *et al.*, 2001). In the latter case, the strain requires transformation with a plasmid encoding a functional HDR for viability. We transformed competent cells made from this strain using both melon HDR isoforms as well as the *E. coli* HDR as a positive control. Once transferred to 0.2% glucose media, this mutant strain grew normally when transformed with either *CmHDR1* or *CmHDR2* (or with the positive control) but not when transformed with *CmDXL* (here used as a negative control) (Fig. 2C). These results demonstrate that both CmHDR1 and CmHDR2 isoforms are active HDR enzymes, in agreement with previous reports of HDR paralogous pairs from gymnosperms (Kim *et al.*, 2008).

### Differential expression of transcripts for different DXS and HDR isoforms suggests functional divergence

**Fig. 4.**
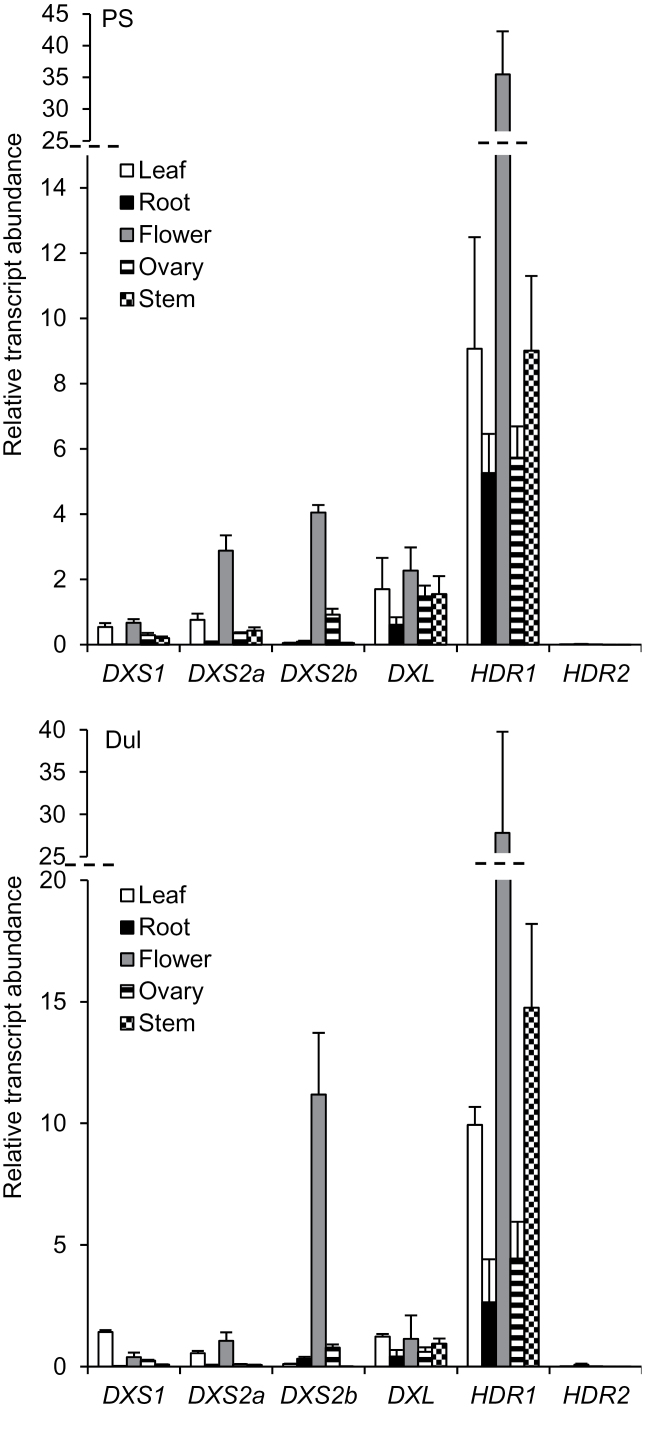
Relative transcript abundance of DXS and HDR isoforms in organs of adult PS (top) or Dul (bottom) plants. cDNA loading was normalized using three distinct reference genes (APT1, ACT2/8, and CYC). RNA was extracted from three individual plants for each organ (*n* = 3) and assayed by quantitative RT-PCR using SYBR Green as three technical replicates each. Relative fold-change calculations were performed using the ΔΔCt model (Livak and Schmittgen, 2001) by normalizing all measurements to the median gene expression in the leaf for all DXS and HDR genes. The dashed line signifies a discontinuity in the vertical axis. Error bars indicate the standard error.

To address the possibility of functional divergence among melon DXS and HDR isoforms, we first carried out a comprehensive characterization of their expression patterns in different organs of adult melon plants from pale and orange varieties. In order to enable comparison among isoforms, fold-change calculations were calibrated to a common median expression level of *DXS* and *HDR* genes. In the pale-fleshed PS variety, *CmDXS1* was expressed in all organs analysed with the exception of roots. By contrast, transcripts for both type II DXS isoforms, *CmDXS2a* and *CmDXS2b*, were most abundant in flowers (Fig. 4). The *CmDXL* isoform showed high transcript levels in all organs, including roots. In the case of HDR-encoding genes, *CmHDR2* expression was almost undetectable, whereas *CmHDR1* transcript levels were much higher than those of any DXS isoform in all organs tested (Fig. 4). The particularly high levels of *CmHDR1* transcripts in flowers, similar to that observed for *CmDXS2a* and *CmDXS2b*, suggest that plastidial isoprenoids produced in melon flowers (mainly carotenoid pigments) require CmHDR1 and type II DXS isoforms at high levels. These expression data are consistent with organ-specific functional specialization of different isoforms of MEP-pathway genes. We compared this data to organ specific expression in the orange-fleshed melon variety Dul, a close relative of Ved. It resembled PS in most respects, such as expression levels of *CmDXS2b* in flower tissue far above that of leaf tissue (Fig. 4). *CmHDR2* expression was absent in most tissues. While low compared to other DXS and HDR genes (Fig. 4), its expression in roots was nonetheless detectable. This stood in contrast to *CmHDR2* expression in PS tissues, in which *CmHDR2* transcripts were completely absent from all organs surveyed.

**Fig. 5.**
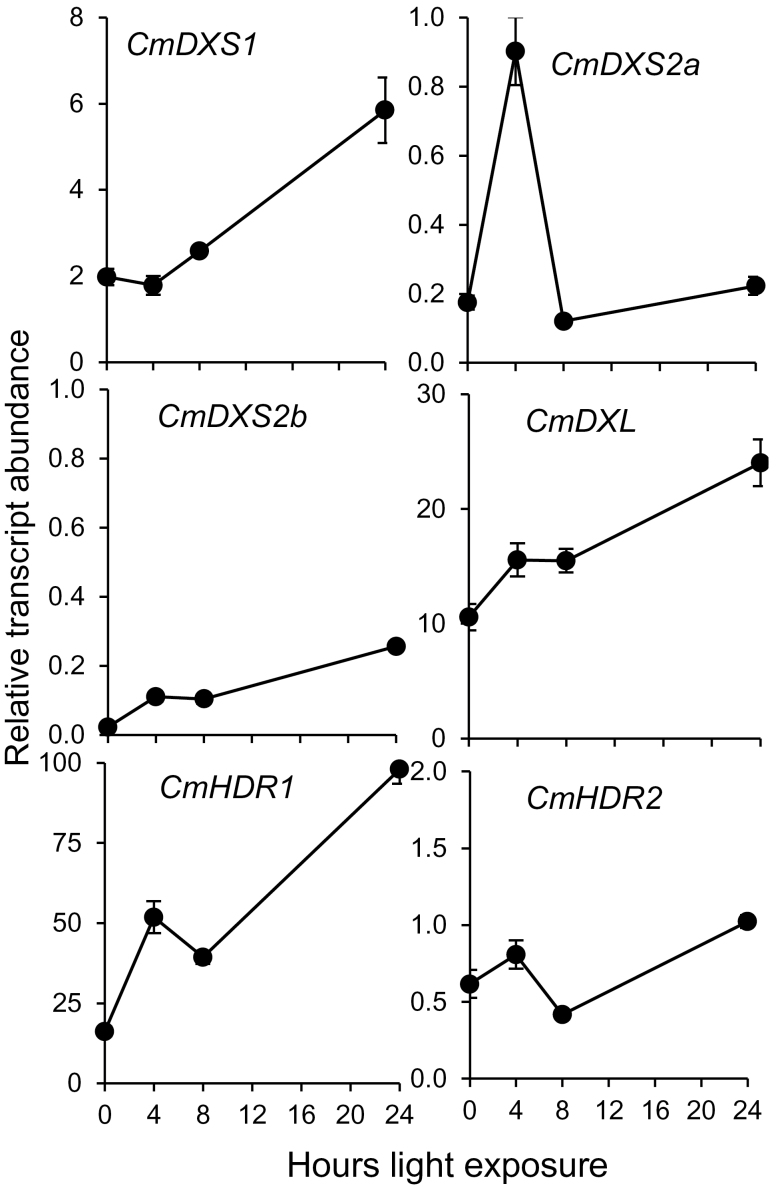
Transcript profiling of etiolated seedlings exposed to white light for 4, 8, or 24h. For each time point, RNA was harvested from three individual seedlings and converted to cDNA as described in Materials and Methods. Quantitative RT-PCR was performed using SYBR Green assays, and cDNA loading was normalized using three separate normalizer genes (APT1, ACT2/8, and CYC). Fold-change calculations were calibrated to the mean expression level of all DXS and HDR genes using the ΔΔCt method (Livak and Schmittgen, 2001). Values shown are the averages of three independent replicate assays in three technical replicates. Error bars indicate the standard error.

De-etiolation (greening) is a major physiological transition to photosynthetic development which requires copious amounts of isoprenoid precursors. We further investigated the differential expression of specific DXS and HDR isoforms by examining their transcript levels in 4-day-old etiolated seedlings at various time points following exposure to continuous white light. Expression levels of *CmDXS1* and *CmDXL* were considerably higher than their counterparts *CmDXS2a* and *CmDXS2b* both before and during light induction (Fig. 5), reaching approximately 6- and 24-fold higher transcript levels at 24h (fully de-etiolated seedlings) relative to those at 0h (etiolated seedlings). This observation suggests that CmDXS1 is the main DXS isoform providing isoprenoid precursors for the transition to photosynthetic development, consistent with previously reported roles of type I DXS isoforms supplying isoprenoid equivalents for photosynthetic metabolism (Walter *et al.*, 2002). In the case of HDR, *CmHDR1* transcripts were the most abundant and the only ones that showed a substantial upregulation upon illumination (Fig. 5), although low levels of *CmHDR2* transcripts were also detected. CmHDR1 supplies IPP and DMAPP for the production of isoprenoids in flowers (Fig. 4) and de-etiolating seedlings (Fig. 5), whereas CmHDR2 may only be expressed in specialized contexts.

### HDR1 and type II DXS isoforms are involved in the production of carotenoids in melon fruit

**Fig. 6.**
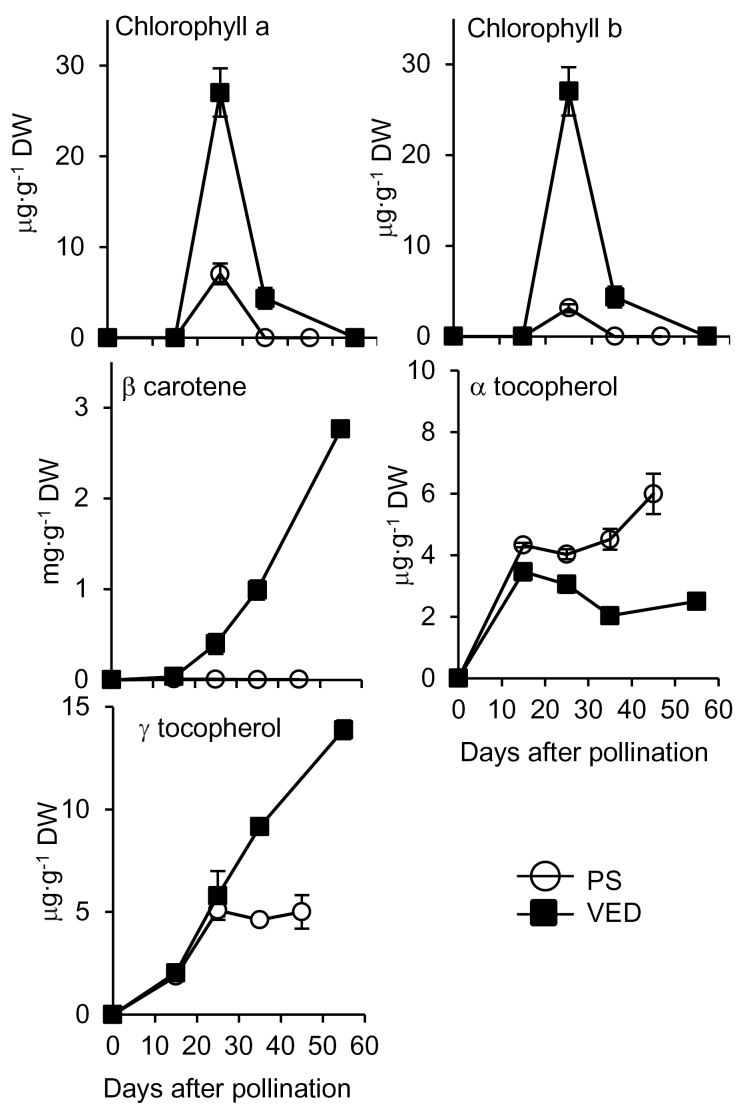
Time course of isoprenoid end-product accumulation in PS and Ved fruit. Developing melon fruit were sampled on the days indicated (three technical replicates from three individual fruits per time point) and extracted. Isoprenoid metabolites in these extracts were separated by HPLC and quantified by comparing their DAD (for chlorophylls and carotenoids) or FLR (for tocopherols) signals to external calibration curves constructed with authentic standards. A chlorophyll *b* standard curve was applied to both chlorophylls and α-tocopherol was similarly employed to quantify both tocopherols. Error bars indicated the standard error of three biological replicates extracted in triplicate. Recoveries were calculated based on individual recoveries of the internal standard canthaxanthin added during the initial extraction step.

To confirm whether specific isoforms might be involved in the production of MEP-derived isoprenoids in certain organs or fruit developmental stages, we next compared the expression of genes encoding DXS and HDR isoforms with the accumulation of different plastidial isoprenoid end-products during melon fruit development in PS and Ved varieties, which produce white and orange-fleshed (carotenoid-accumulating) fruits, respectively, when ripe. As shown in Fig. 6, analysis of MEP-derived isoprenoid end-products in developing melon fruit by HPLC-DAD-FLR revealed major differences between PS and Ved in the production of carotenoids (mainly β-carotene) and tocopherols. Only low levels of β-carotene were detected early in PS fruit development, and these decreased to barely detectable levels towards fruit maturation and ripening (Fig. 6). In contrast, fruit ripening in Ved was accompanied by a dramatic accumulation of β-carotene, reaching 2.76±0.05 mg·g^–1^ DW by harvest. Compared to immature fruit (15 DAP), this corresponds to a nearly 80-fold increase. Chlorophyll *a* and *b* were present at low levels at earlier stages of fruit development of both strains and detected only intermittently thereafter. In ripe fruit, very low levels of chlorophylls remained in both varieties (Fig. 6). In the case of tocopherols, both α and γ tocopherol increased as fruit developed but with different profiles in PS and Ved (Fig. 6). The level of tocopherols increased in parallel over time up to 15 DAP (α) or 25 DAP (γ) in both varieties. From that stage, α-tocopherol modestly increased in ripening PS fruit whereas γ-tocopherol substantially increased in the Ved variety. As a result, ripe PS fruit accumulated roughly equal amounts of α- and γ-tocopherol (5–6 μg·g^–1^ DW) whereas Ved fruit contained almost three times more γ-tocopherol than α-tocopherol (Fig. 6). In terms of total tocopherol content, Ved had approximately 50% more tocopherols than PS (16.4 vs 11.0 μg·g^–1^ DW).

**Fig. 7.**
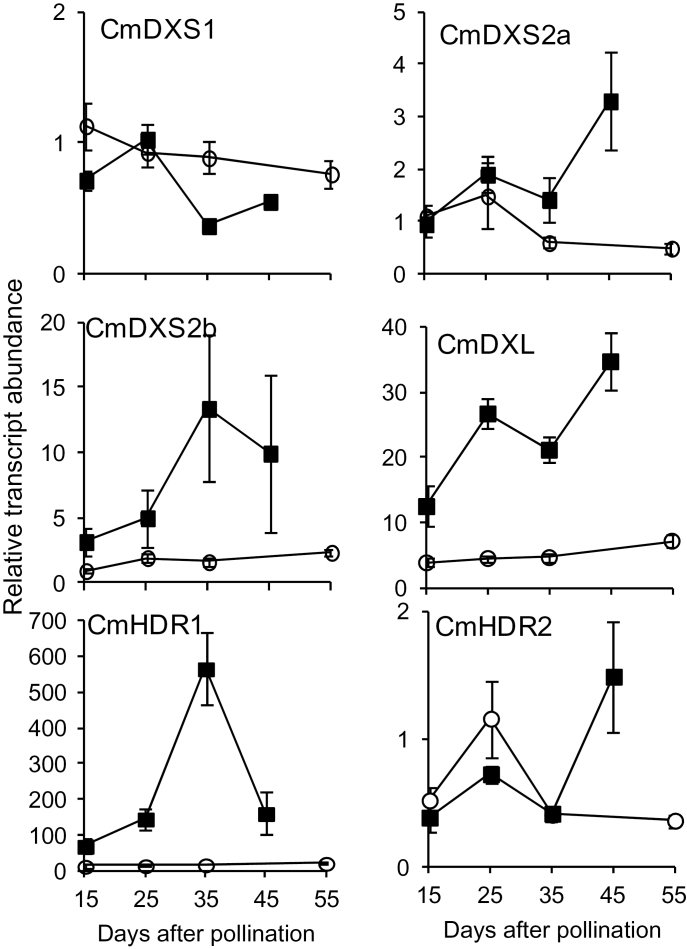
Transcript profiling in developing melon fruit. Quantitative RT-PCR SYBR Green assays were performed at different development fruit stages for PS (open circles) or Ved (filled squares) fruit. RNA was extracted from samples from three individual melons. cDNA loading was normalized using the CYC, APT1, and ACT2/8 genes. Relative fold calculations were based on the median gene expression of all DXS and HDR genes from a given variety using the ΔΔCt method (Livak and Schmittgen, 2001). Values shown are the averages of three biological replicates assayed in triplicate. Error bars represent the standard error.

When the expression of DXS and HDR family genes was analysed in developing PS and Ved fruit, no induction of *CmDXS1* or *CmHDR2* was observed during ripening for either cultivar (Fig. 7). By contrast, the rest of the genes analysed (*CmDXS2a*, *CmDXS2b*, *CmDXL*, and *CmHDR1*) were upregulated during fruit development in Ved but not in PS. Because CmDXL was found to be inactive as a DXS enzyme (Fig. 2), we conclude that CmDXS2a, CmDXS2b, and CmHDR1 are most likely the isoforms involved in supplying the precursors required not only for carotenoid (β-carotene) but also for γ-tocopherol production during orange-fleshed Ved fruit ripening.

To determine if *cis*-acting regulatory elements could account for the observed transcript differences between Ved and PS, we used whole genome re-sequence data to extract the promoter (2kb) and 3ʹ regions (1kb) of the these genes (*CmDXS2a*, *CmDXS2b*, and *CmHDR1*) for each variety (PS and Ved). We observed no differences in *CmHDR1 cis* regulatory regions when PS and Ved were compared, implying that regulation by *trans*-acting elements is at least partially responsible for the observed induction in Ved. We likewise observed no differences in the untranslated regions or predicted transcriptional start sites of *CmDXS2a* or *CmDXS2b* when PS and Ved were compared. However, a comparison of the promoter regions between varieties revealed subtle differences for both *CmDXS2a* and *CmDXS2b*, consisting of 12 SNPs and a 3-bp deletion in Ved in the case of *CmDXS2a* and 15 SNPs and a 3-bp deletion in Ved in the case of *CmDXS2b*. An analysis of known transcription factor binding sites revealed many differences based on these nucleotides changes. A full listing of transcription factor binding site differences between varieties for these two genes is provided in Supplementary Table 2. BOXIINTPATPB and POLLEN1LELAT52 represent the only motifs present in the promoter regions of both *CmDXS2a* and *CmDXS2b* of Ved that are absent in PS. While these differences could in principal account for the differences in transcriptional regulation of type II DXS genes between white- and orange-fleshed varieties, the lack of differences in *cis* elements of *CmHDR1* suggests that carotenoid formation in melon fruit is a complex process with multiple regulatory mechanisms at work.

## Discussion

### Melon DXS type I and II family members are functionally specialized for primary and secondary isoprenoid production, respectively

DXS is represented as a small gene family in most plant species, and here we have investigated the roles of the four melon genes encoding proteins with high similarity to known DXS proteins: one type I (*CmDXS1*), two type II (*CmDXS2a* and *CmDXS2b*), and one type III (Cm*DXL*). While *CmDXS1*, *CmDXS2a*, and *CmDXS2b* encode functional DXS proteins, *CmDXL* does not. A phylogenetic tree describing the evolutionary relationship of known plant DXS proteins shows that the melon DXS proteins group within their expected type I, II, or III (DXS-like) categories rather than with themselves, in keeping with the general observation that duplication and functional specialization of these genes preceded the separation of the major plant lineages (Walter *et al.*, 2002). The phylogenetic evidence also suggests that distinct subclasses within type II DXS isoforms are a genuine feature within the type II DXS clade (Fig. 1A–D). Based on the hierarchical organization of DXS protein sequences, the duplication of type II DXS genes may have occurred on multiple occasions. Unexpectedly, angiosperm sequences of clade A (Fig. 1) are more closely related to the gymnosperm type II proteins of clade C than to their own intraspecific paralogues, and a similar relationship for gymnosperm type II DXS paralogues exists. This could be explained by the duplication of type II DXS genes both before and after the separation of angiosperm and gymnosperm lines. Additional research is necessary to determine whether clade B is an evolutionarily recent offshoot from the common branch giving rise to angiosperm and gymnosperm type II DXS genes (Fig. 1, clades A and C). While transcript profiling did not suggest functional specialization of *CmDXS2a* relative to *CmDXS2b*, the hierarchical grouping of these sequences into classes, rather than forming their own clade, suggests functional divergence is under way.

*CmDXS1* is induced by light and is highly expressed in leaves and flowers, which is consistent with a role in providing isoprenoid equivalents for primary metabolism (i.e. photosynthesis). *CmDXS2a* and *CmDXS2b*, however, are primarily expressed in flower, ovaries, and developing fruit. Based on a time course to observe the accumulation of β-carotene in Ved fruit following pollination, the rate of accumulation of this compound increased considerably after 15 DAP and then continued to increase until harvest (Fig. 6). The white-fleshed variety PS, in contrast, accumulates only trace levels of chlorophylls or carotenes (Fig. 6). CmDXS2a and CmDXS2b are the dominant DXS isoforms in developing fruit of orange varieties, and hence they are probably involved in providing precursors for the high levels of plastidial isoprenoids (including β-carotene and, to a much lower extent, γ-tocopherol) that accumulate during ripening. In contrast, neither of these type II *DXS* transcripts is upregulated during fruit development in the PS variety which lacks β-carotene, implying that elevated DXS activity is only necessary in varieties which accumulate high amounts of carotenoid end products. We identified two *cis*-regulatory elements present in the promoters of Ved *CmDXS2a* and *CmDXS2b* which were absent in the promoters of the corresponding PS genes: BOXIINTPATPB, a non-consensus type II promoter of plastid genes identified in tobacco (Kapoor and Sugiura, 1999) and POLLEN1LELAT52, a motif required for pollen-specific expression in tomato (Bate and Twell, 1998) (Supplementary Table 2). Curiously, carotenoid cleavage dioxygenase (CmCCD1), which produces volatile apocarotenoids from β-carotene through a 9,10-oxidative cleavage, is upregulated both in orange-fleshed varieties as well as in white varieties that accumulate neither carotenoids nor apocarotenoids (Ibdah *et al.*, 2006). This suggests the transcriptional programme for β-carotene catabolism is uncoupled from that of precursor production via the MEP pathway.

Type II DXS isoforms in other plant species have been implicated in defensive oleoresin production (Phillips *et al.*, 2007), ginkgolide natural product formation (Kim *et al.*, 2006), and apocarotenoid production in association with arbuscular mycorrhizas (Walter *et al.*, 2000), but this is the first report of a type II DXS involved in specialized isoprenoid biosynthesis in the fruit of an agronomically important crop species such as melon. *Citrus*, for instance, relies on a type I DXS for carotenoid formation during fruit ripening (Peng *et al.*, 2013). In tomato, the best characterized system for fleshy fruit-producing agricultural crops, a type I DXS isoform also appears to supply the precursors for carotenoid biosynthesis during fruit ripening whereas the type II enzyme is expressed preferentially in trichomes (Lois *et al.*, 2000; Paetzold *et al.*, 2010).

### DXL expression correlates with isoprenoid biosynthesis but does not encode a functional DXS protein

**Fig. 8.**
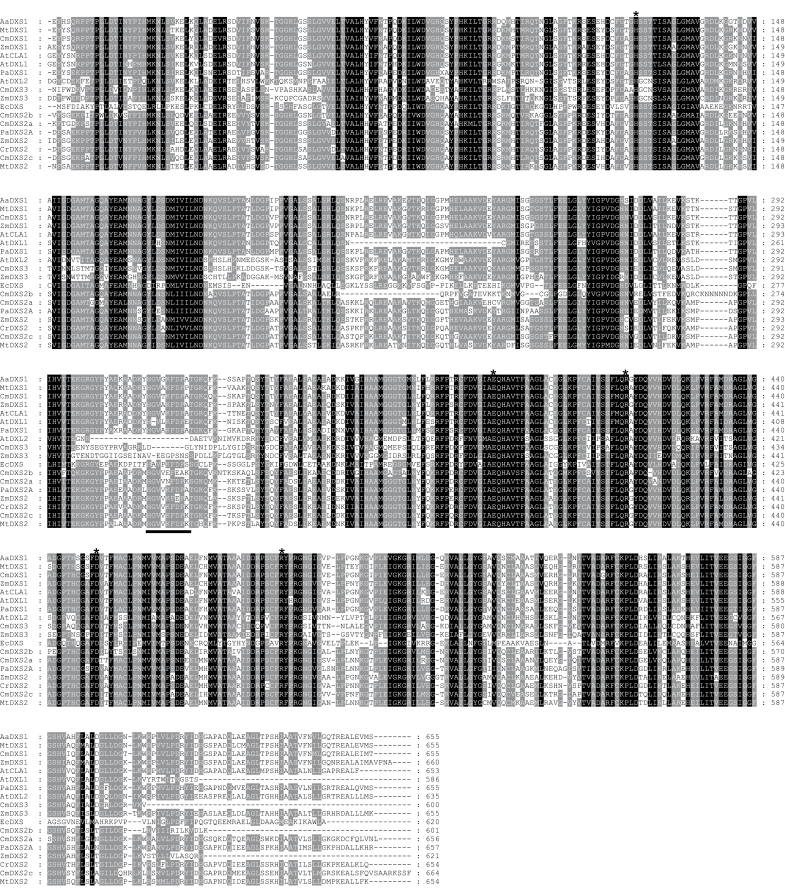
Alignment of selected type I and II DXS sequences. The alignment was created using BLOSSUM62 with a gap-opening penalty of 10 and a gap-extension penalty of 0.05. It includes the presumed mature peptide only (transit peptides were manually curated based on an alignment of the full-length peptide). Residues shaded in black are absolutely conserved while those in grey are conserved among 70% of sequences analysed. The solid bar indicates a deletion in type III DXS sequences. Asterisks indicate residues identified as critical for catalysis in the *E. coli* protein (Xiang, *et al.* 2007). The first two letters of each sequence indicates the corresponding species: Aa, *Artemisia annua*; Mt, *Medicago truncatula*; Cm, *Cucumis melo*; Zm, *Zea mays*; At, *Arabidopsis thaliana*; Pa, *Picea abies*; Ec, *Escherichia coli*; Cr, *Catharanthus roseus*; CmDXS3, melon DXL.

*CmDXL* was induced during de-etiolation in Ved (Fig. 5) and during fruit ripening in Ved but not PS (Fig. 7). The failure to detect authentic DXS activity in CmDXL by two independent methods, coupled to its highly upregulated transcripts in developing fruit and de-etiolating seedlings, suggests it encodes a protein with an activity different from that of DXS. *Arabidopsis* AtDXL1 (a type I DXS) was found to be a non-essential gene which similarly lacks DXS activity (Carretero-Paulet *et al.*, 2013). Unlike its functional paralogue CLA1/DXS1, it is not expressed in leaves or induced by light and is mainly expressed in siliques and flowers. An evolutionary analysis of this plastid-localized protein suggested it had evolved recently through gene duplication of CLA1/DXS1 in the Brassicaceae line (Carretero-Paulet *et al.*, 2013). The additional observation that CmDXL suppresses endogenous DXS activity in *in vitro* assays of crude protein preparations (compared to non-transformed controls) suggests it may share a common substrate with DXS. In that event, CmDXL would effectively mask endogenous activity *in vitro* due to its affinity for a substrate required by DXS. An analysis of the CmDXL amino acid sequence shows that it contains many typical features of a DXS enzyme, including a thiamin diphosphate co-factor binding domain, a transketolase domain, and essential amino acids implicated in the DXS mechanism (His^192^, Asp^445^, Glu^379^, Arg^407^, and Arg^487^; Fig. 8) (Xiang *et al.*, 2007). However, amino acids 182–195 of CmDXL depart sharply from the conserved motif in the corresponding position of type I and II DXS sequences, and this motif is also divergent in maize ZmDXS3 and *Arabidopsis* AtDXL2 (Fig. 8). CmDXL also lacks a conserved HGVXKFPD motif present in DXS proteins of classes I and II (corresponding to amino acids 377–384 of CmDXS1). In another type III clade member, *Arabidopsis* DXL2, this motif is also absent, and in maize ZmDXS3 it is substituted by an unrelated sequence (Fig. 8). Based on the *E. coli* DXS 3D structure, the first residue of this motif (His^304^ in the *E. coli* protein) is predicted to participate in binding of glyceraldehyde 3-phosphate. Given the absence of this conserved residue in CmDXL, an inability to bind glyceraldehyde 3-phosphate could explain its lack of DXS activity. The expression of *ZmDXS3* is linked to carotenoid accumulation in maize endosperm (Vallabhaneni and Wurtzel, 2009), and a plasmid bearing this gene was capable of rescuing a *dxs*-deficient *E. coli* strain when grown in liquid culture (Cordoba *et al.*, 2011). However, this approach may also favour spontaneous recruitment of DXS activity from other genes (Sauret-Gueto *et al.*, 2003; Sauret-Gueto *et al.*, 2006; Perez-Gil *et al.*, 2012). Additional research is required to definitively assign a biochemical function to members of this clade.

### The mode of HDR paralogue acquisition differs across plant lineages and impacts subfunctionalization

The melon genome possesses two functional HDR genes, one of which, *CmHDR1*, is expressed at high levels in most tissues and developmental processes. *CmHDR2* transcripts were only detected in de-etiolating cotyledons and in roots of orange-fleshed varieties. However, a similar pattern of transcript expression in young leaves and roots was reported for *Ginkgo biloba GbHDR2* (Kang *et al.*, 2013). This supported previous transcript profiling of MEP-pathway genes in cultured gingko embryos (Kim *et al.*, 2008) and *Pinus densiflora* (Kim *et al.*, 2009) which suggested a specialized role for gymnosperm HDR2 proteins in specialized isoprenoid biosynthesis; for example, gingkolide biosynthesis in roots of *G. biloba* and diterpenoid resin acid biosynthesis in pine species. Phylogenetic analysis also supported the existence of subclades of HDR which reflect an ancient functional specialization of HDR paralogs (Kim *et al.*, 2008). We observed that the ancient division yielding HDR paralogues in modern gymnosperms (Fig. 3A and B) described by Kim *et al.* (2008) was not fully preserved in angiosperms. Instead, members of HDR clade B share a common ancestor with all angiospermous HDR sequences. Further divisions of this group have occurred independently in some flowering plant families, leading to divergent HDR copies in the Cucurbitaceae (Fig. 3C and D). However, this analysis also revealed many angiosperm lines, such as apple, soybean, rape, cotton, banana, and poplar, with two highly similar copies of HDR (Fig. 3 and inset) and no indication of functional divergence which could be inferred by sequence relatedness. Unlike the clear hierarchical division of DXS types, the phylogeny of HDR suggests at least two distinct legacies for HDR paralogue acquisition are represented here.

We considered the role of large-scale genome duplication events in the duplication of MEP-pathway genes and observed an absolute correlation between species with highly similar HDR paralogues, including banana, soybean, poplar, rape, and apple, and a history of polyploidy. In these species, HDR paralogues most likely represent homoeologous sequences produced by large scale or WGD events. On the other hand, HDR paralogues which arose in species with no recent record of genome duplications, such as the cucurbits (Garcia-Mas *et al.*, 2012) and gymnosperms (Nystedt *et al.*, 2013), are probably products of tandem or segmental duplications. These small-scale duplications can lead to subfunctionalization more rapidly than duplications derived from WGD events (Flagel and Wendel, 2009). The duplication-degeneration-complementation (DDC) model of subfunctionalization posits that duplicates arising from an ancestral gene with multiple functions (including multiple expression domains) may become quickly specialized by accumulating mutations which partition the ancestral functions such that both duplicates acquire essential roles (Force *et al.*, 1999), resulting in the preservation of both genes. Many models of gene duplication and evolution have been proposed, and multiple modes may act in concert to preserve duplicate genes (Innan and Kondrashov, 2010). For example, escape from adaptive conflict (EAC) (Des Marais and Rausher, 2008) could also explain the origin of specialized HDR paralogues if multiple functions performed by the original gene could not be improved upon independently. While a combination of subfunctionalization, DDC, and EAC may explain retention of specialized HDR paralogues in species without a history of WGDs, the existence of such homoeologues in banana, apple, poplar, rape, and soybean is perhaps more difficult to explain considering WGD should result in multiple copies of upstream steps as well.

The observation that only the first and last steps of the MEP pathway are frequently encoded in the genome as gene families may be explained by gene dosage imbalance. Briefly, this hypothesis maintains that stoichiometric imbalances in macromolecular complexes can lead to dosage-dependent phenotypes (Veitia *et al.*, 2008). Gene balance can determine which genes are preferentially retained after single gene duplications or WGD events. Overexpression (via gene duplication) may be harmful if that gene product forms a multi-subunit complex. A high number of subunits in a complex increases the probability that the members are encoded by singleton genes (Papp *et al.*, 2003; Yang *et al.*, 2003). In such cases, WGDs are followed by gene loss and pseudogenization. The existence of multi-enzyme complexes formed by enzymes of the MEP pathway has not been studied in plants. However, in some bacterial species, central steps of the MEP pathway (but not DXS or HDR) do form such complexes (Gabrielsen *et al.*, 2004; Lherbet *et al.*, 2006). If similar complexes exist in plants, gene dosage balance may be responsible for the loss of multiple copies of these central steps while multiple copies of DXS and HDR are retained in some (but not all) cases.

Here we have described the role of specialized isoforms encoding regulated steps of the MEP pathway in melon and provided evidence for their functional specialization in seedling greening and fruit development. Understanding the regulation of carotene accumulation in melon fruit flesh is important for developing new tools for breeding programmes in this species, and we have identified *CmDXS2a*, *CmDXS2b*, and *CmHDR1* as genes important for fruit colour and quality. We have also demonstrated that CmDXL, a member of the type III DXS clade, is not a genuine DXS. These results contribute to a better understanding of evolution of the MEP pathway in the following ways. First, our findings indicate that in addition to an ancient separation of DXS into functional classes, further duplications and divergence within the type II clade have taken place. Second, we have shown that the history of HDR gene duplication is very different from that of DXS. We identified two disparate modes of HDR duplicate acquisition in plants: ancient small-scale gene duplications in the cases of cucurbits and gymnosperms and more recent polyploidy in the case of other flowering plant families. These differences may partially explain differences in the isoprenoid regulatory programmes of extant plants. The evolutionary history of a plant species may have included WGDs, gene loss, pseudogenization, single gene duplications, and functional specialization, and here we have explored the impact of such events on the regulation of the MEP pathway in melon. Continued research in this area will provide new insights into the regulatory programme for the biosynthesis of this important class of natural products.

## Supplementary material

Supplementary data can be found at *JXB* online.

Supplementary Table S1. Primers used in the study.

Supplementary Table S2. Occurrences of *cis*-regulatory elements in the promoters of type II DXS sequences of PS and Ved melon varieties.

## Funding

MP and MS were supported by a JAE-Doc grant from the Spanish Ministry of Science. MP was also supported by a post-doctoral fellowship from the Deutsche Forschungsgemeinschaft (PH 179/1-1) and a Ramon y Cajal contract from the Spanish Ministry of Science. This work was funded by grants from the Spanish Dirección General de Investigación (BIO2011-23680 and PIM2010IPO-00660), Programa Iberoamericano de Ciencia y Tecnologia para el Desarrollo (IBERCAROT) (to MRC), the Fundación Genoma España (MELONOMICS project), and the Spanish Ministry of Science and Innovation (CSD2007-00036 and GEN2006-27773-C2-1) (to JGM).

## Supplementary Material

Supplementary Data
