# Supplementary material for: The 2-C-methylerythritol 4-phosphate pathway in melon is regulated by specialized isoforms for the first and last steps
Source: J Exp Bot. 2014 Jul 10;65(17):5077–92. doi: 10.1093/jxb/eru275 (PMC4144782; doi:10.1093/jxb/eru275)
Supplement: Supplementary Data [file supp_65_17_5077__index.html]

The 2-C-methylerythritol 4-phosphate pathway in melon is regulated by specialized isoforms for the first and last steps — The 2-C-methylerythritol 4-phosphate pathway in melon is regulated by specialized isoforms for the first and last steps — Supplementary Data 

# The 2-*C*-methylerythritol 4-phosphate pathway in melon is regulated by specialized isoforms for the first and last steps

## Supplementary Data

Data files

**Files in this Data Supplement:**

- Supplementary Data - Supplementary Data
