# Supplementary material for: The 2-C-methylerythritol 4-phosphate pathway in melon is regulated by specialized isoforms for the first and last steps
Source: J Exp Bot. 2014 Jul 10;65(17):5077–92. doi: 10.1093/jxb/eru275 (PMC4144782; doi:10.1093/jxb/eru275)
Supplement: Supplementary Data [file supp_eru275_jexbot127076_file001.pdf]

Supplementary tables for the following manuscript

"The 2-*C*-methylerythritol 4-phosphate pathway in melon is regulated by specialized isoforms for the first and last steps"

Montserrat Saladié[a†], Louwrance P. Wright[b], Jordi Garcia-Mas[a], Manuel Rodriguez Concepcion[c], Michael A. Phillips[c*]

[a] Plant and Animal Genomics Program, Institut de Recerca i Tecnologia Agroalimentària and Centre for Research in Agricultural Genomics, CSIC-IRTA-UAB-UB, 08193 Barcelona, Spain

[b] Department of Biochemistry, Max Planck Institute for Chemical Ecology, Beutenberg Campus, Hans Knöll Str. 8, 07745 Jena, Germany

[c] Plant Metabolism and Metabolic Engineering Program, Center for Research in Agricultural Genomics, CSIC-IRTA-UAB-UB, 08193 Barcelona, Spain

Supplementary table I: Primers used in this study

Primer	Sequence	Purpose
CmDXSA attB1.1	GGGGACAAGTTTGTACAAAAAAGCAGGCTTCTAAGCGATGTCTTCC CACCTTCTCACAAATTC	CmDXS2a Gateway cloning primer
CmDXSA-attB2	GGGGACCACTTTGTACAAGAAAGCTGGGTGTTATAAATTCACAAGT TGAAAGC	CmDXS2a Gateway cloning primer
CmDXSApm attB1	GGGGACAAGTTTGTACAAAAAAGCAGGCTTCATGTTCACGGCAGAC AAGCCATCCACTCCC	CmDXS2a Gateway cloning primer without transit peptide
CmDXSBY-attB1	GGGGACAAGTTTGTACAAAAAAGCAGGCTTCATGAAACCAGCGACC CCATTGTTGGACAC	CmDXS2b Gateway cloning primer
CmDXSBY-attB2	GGGGACCACTTTGTACAAGAAAGCTGGGTGCTAGAAGCTTGACTTC CTAGCTGCC	CmDXS2b Gateway cloning primer
CmDXSBYpm-attB1	GGGGACAAGTTTGTACAAAAAAGCAGGCTTCATGAAACCAGCGACC CCATTGTTGGACAC	CmDXS2b Gateway cloning primer without transit peptide
CmDXSC-attB1	GGGGACAAGTTTGTACAAAAAAGCAGGCTTCATGAGGCCACCAACT CCTCTTTTGG	CmDXS1 Gateway cloning primer
CmDXSC-attB2	GGGGACCACTTTGTACAAGAAAGCTGGGTGCTATGTCATGATCTCA AGAGCTTCC	CmDXS1 Gateway cloning primer
CmDXSCpm attB1	GGGGACAAGTTTGTACAAAAAAGCAGGCTTCATGTATCCTTCACAG AGGCCACCAACTCC	CmDXS1 Gateway cloning primer without transit peptide
CmDXSEpm-attB1	GGGGACAAGTTTGTACAAAAAAGCAGGCTTCATGAAAGTTCCAACG CCTCTACTTGATG	CmDXL Gateway cloning primer without transit peptide
CmDXSE-attB2	GGGGACCACTTTGTACAAGAAAGCTGGGTGTCATACCTTGACTCTTC CATCCAGC	CmDXL Gateway cloning primer
CmDXR attB1	GGGGACAAGTTTGTACAAAAAAGCAGGCTTCGTAATGGCGTTGAAT GTTCTTGC	CmDXR Gateway cloning primer
CmDXR attB2	GGGGACCACTTTGTACAAGAAAGCTGGGTGTTATGCGGCTACGGGA CTAAAAG	CmDXR Gateway cloning primer
CmCMS attB1	GGGGACAAGTTTGTACAAAAAAGCAGGCTTCAAGCCTCGAGATGGC AACTGTAACCCG	CmCMS Gateway cloning primer
CmCMSpm attB1	GGGGACAAGTTTGTACAAAAAAGCAGGCTTCATGATTGTGAAAGAG AGAAGTGTT	CmCMS Gateway cloning primer
CmCMSstp attB2	GGGGACCACTTTGTACAAGAAAGCTGGGTGTCACGAGTTCAAATTC AATATTCTCT	CmCMS Gateway cloning primer without transit peptide
CmCMKpm attB1	GGGGACAAGTTTGTACAAAAAAGCAGGCTTCATGTCGAGGCTTACT CTGTTCTCACC	CmCMK Gateway cloning primer without transit peptide
CmCMKgstp attB2	GGGGACCACTTTGTACAAGAAAGCTGGGTACGCTTTTGTTACATTCA A	CmCMK Gateway cloning primer
CmMDSpm attB1	GGGGACAAGTTTGTACAAAAAAGCAGGCTTCATGTTTCGGGTTGGA CACGGCTTCGATC	CmMDS Gateway cloning primer without transit peptide
CmMDSstp attB2	GGGGACCACTTTGTACAAGAAAGCTGGGTGCTATCTCCTCATCAAC AGGACCAC	CmMDSGateway cloning primer
HDRApm-attB1	GGGGACAAGTTTGTACAAAAAAGCAGGCTCATGGATTTCAATGCGA AGCTGTTCTTG	CmHDR2 Gateway cloning primer without transit peptide
HDRAstp-attB2	GGGGACCACTTTGTACAAGAAAGCTGGGTTTATTATCCAAGCGATT CGAACTGTGC	CmHDR2 Gateway cloning primer
HDRBpm-attB1	GGGGACAAGTTTGTACAAAAAAGCAGGCTCATGGATTTTGACGCCA	CmHDR1 Gateway cloning primer

	AGGTGTTCCGG	without transit peptide
HDRBstp-attB2	GGGGACCACTTTGTACAAGAAAGCTGGGTTTTATTATGCAAACTGC AAAGCTTCCTC	CmHDR1 Gateway cloning primer
CmACTIN28-F	GGCAACATTGTTCTCAGTGGTGG	Actin QPCR primer
CmACTIN28-R	AACGACCTTGATTTTCATGCTGC	Actin QPCR primer
CmAPT1A	GTTGCAGGAATTGAGGCTAGAGGG	APT1 QPCR primer
CmAPT1A-R	TGGCTCCAATAGCCAAAGCAATAG	APT1 QPCR primer
CmCYC-F	CGATGTGGAAATTGACGGAA	Cyclophilin QPCR primer
CmCYC-R	CCTGGAAATTGGGCATGC	Cyclophilin QPCR primer
DXSA-1F	CAAATTGATGAGCAATGCTTT	CmDXS2a QPCR primer
DXSA-1R	AGAAAAGAATTTGTGAGAAGGTG	CmDXS2a QPCR primer
DXSBY-1F	GCTTCTCATCAAACCAACTCAA	CmDXS2b QPCR primer
DXSBY-1R	TTCTTTATCTGGCAAATTCTTCC	CmDXS2b QPCR primer
DXSC-1F	GCTCTGTATGCTAACTTTTCCTG	CmDXS1 QPCR primer
DXSC-1R	GAAACAGCAACACTTGAACCA	CmDXS1 QPCR primer
CmDXSE-1F	GGCCACAATATCGAGGACC	CmDXL QPCR primer
CmDXSE-1R	CCCTCGGGTAACCTTCTGA	CmDXLQPCR primer
HDRA-F	ACGAGCTGGTGAAGGAAGATGT	CmHDR2 QPCR primer
HDRA-R	GTGAGCGAGCTTGTATGATATTGAG	CmHDR2 QPCR primer
HDRB-F	ACTTGAGGAAGGAAGGAAACAGTTT	CmHDR1 QPCR primer
HDRB-R	TGTCAACATCTCATCCACCGC	CmHDR1 QPCR primer

Supplementary table II: Occurrences of *cis*-regulatory elements in the promoters of type II DXS sequences of white-fleshed (PS) and orange-fleshed (Ved) melon varities

		DXS2a		DXS2b		
Motif	Sequence	PS	Ved	PS	Ved	Putative function
ABRELATERD1	ACGTG	3	3	1	0	Dehydration/ABA-responsive
ABRERATCAL	MACGYGB	1	1	1	0	Ca2+-responsive
ACGTATERD1	ACGT	8	8	6	4	Dehydration/ABA-responsive
AMYBOX1	TAACARA	1	1	1	0	Amylase box
ARR1AT	NGATT	18	18	13	12	ARR response
BOXIINTPATPB	ATAGAA	2	3	2	3	NCII
DOFCOREZM	AAAG	27	28	41	40	DOF binding site
EBOXBNNAPA	CANNTG	14	14	14	12	Storage protein
GAREAT	TAACAAR	1	1	1	0	GA-responsive
GATABOX	GATA	19	19	18	20	Light responsive
GT1CONSENSUS	GRWAAW	28	28	24	26	Light responsive
GTGANTG10	GTGA	10	10	9	10	Pollen specific
IBOX	GATAAG	2	2	1	0	Light responsive
IBOXCORE	GATAA	6	6	5	6	Light responsive
MYBGAHV	TAACAAA	1	1	1	0	GA-responsive
MYCCONSENSUSAT	CANNTG	14	14	14	12	Dehydration responsive
POLASIG1	AATAAA	17	16	6	6	Polyadenylation signal
POLLEN1LELAT52	AGAAA	13	14	20	21	Pollen specific
PROLAMINBOXOSG LUB1	TGCAAAG	0	0	2	1	Seed/endosperm specific
PYRIMIDINEBOXOS RAMY1A	CCTTTT	3	3	5	4	GA-responsive
QARBNEXTA	AACGTGT	1	1	1	0	Extensin
SEF4MOTIFGM7S	RTTTTTR	7	7	6	5	Seed storage
SURE1STPAT21	AATAGAAA	0	0	0	1	Sucrose responsive element
T/GBOXATPIN2	AACGTG	1	1	1	0	JA-responsive
TATABOX5	TTATTT	21	20	5	5	TATA box
TBOXATGAPB	ACTTTG	2	2	3	2	Light responsive
WUSATAg	TTAATGG	1	1	1	1	WUS target sequence
